# Red flags for the differential diagnosis of granulomatous mastitis: a case report

**DOI:** 10.1186/s13256-020-02563-x

**Published:** 2020-11-10

**Authors:** Richard Chalmers, Patrick McClellan, Vixey Silva, Natalie Shutt, Carolina Restini

**Affiliations:** 1 Family Medicine Residency Program, McLaren Macomb Hospital, 1000 Harrington Blvd, Mount Clemens, MI 48043 USA; 2 Department of Pharmacology and Toxicology, College of Osteopathic Medicine, Michigan State University, 44575 Garfield Road, Building UC4., Clinton Township, MI 48038 USA

**Keywords:** Granulomatous mastitis, Case report, Rare breast disease, Chronic inflammation, Red flags, Multi-professional health care

## Abstract

**Background:**

Granulomatous mastitis (GM) is a rare benign chronic inflammatory breast disease. GM presents as a heterogeneous illness with variable clinical presentations, and its diagnosis is usually made by exclusion. There are no guidelines for the treatment of GM. This manuscript describes the management of a patient with GM, initially unsuccessfully treated outside our clinic under a diagnosis of mastitis. The patient’s history, physical examination, and needle biopsy flagged the patient’s findings as nonmalignant; however, imaging studies indicated a tumor. Differential diagnosis became a critical element of her care. This case report represents a valuable resource to foster more assertive clinical practice in managing patients with GM. The case coordination and its course were led by a team from an outreach clinic that provides health care services to underserved communities in the state of Michigan.

**Case presentation:**

A 41-year-old G1P1 Hispanic female immigrant from Central America presented with a rare breast disease, granulomatous mastitis. A similar presentation occurred 5 years before pregnancy when she had an episode of pain and swelling in the left breast, which resolved spontaneously. She sought our services after being diagnosed with mastitis that was unsuccessfully treated. Physical examination revealed a nodular mass in the outer quadrants of the left breast without regional lymphadenopathy. Needle biopsy showed fibrohistiocytic and florid inflammatory reactions, with no evidence of invasive carcinoma. However, this result was inconsistent with the degree of abnormality revealed by the mammogram (BI-RADS grade 5), ultrasound, and physical examination. Full incisional biopsy revealed cystic neutrophilic GM. The surgical procedure, antibiotics, and corticosteroids resulted in a successful combination to secure the stable control of the symptoms and progression of this rare benign breast disease to date.

**Conclusions:**

This patient’s case highlights the importance of integrated communication among front-line primary care and other health care professionals to reduce the risk of invasive procedures and avoid institutional costs. GM is a rare disease. We raised the manifold red flags in which the multiple professional chains recruited to care for this patient were concerning for advanced breast cancer. The lack of experience and evidence-based medicine contributed to the contradictory interpretation of the findings on GM's diagnosis.

## Background

We report a case of granulomatous mastitis, an unusual presentation of breast disease.

Granulomatous mastitis (GM) is an uncommon benign chronic inflammatory breast disease first described by Kessler and Wolloch in 1972 [1] that can mimic inflammatory breast cancer and periductal mastitis [2]. It typically affects young women between 17 and 42 years of age within the reproductive and post-childbearing period. GM constitutes 24% of all inflammatory breast diseases [3]. The etiology of GM is unclear. Postulated factors include infectious or autoimmune diseases, immunologic response to milk leakage from the breasts’ lobules [4, 5], and reaction to oral contraceptives. Granulomas are usually found around lobules and ducts of the breast in the absence of specific infection, trauma, or evidence of sarcoidosis [6].

GM presents as a heterogeneous disease with variable clinical presentations [7], and the diagnosis is made by exclusion. There are no guidelines for the treatment of GM. This case report represents a valuable resource to foster more assertive clinical practice in managing patients with GM. The case coordination and its course were led by a team from an outreach clinic that provides health care service to underserved communities, in partnership with a multidisciplinary team from the Family Practice Residency Program in a community hospital, in the state of Michigan.

Based on our experience, we aim to raise the red flags relevant to identifying contradictory findings in the course of diagnosis, and to also draw attention to potential adverse reactions during treatment.

### Contextualizing the health care attention provided to the reported patient

The case was conducted by the Medical Outreach Clinic (MOC) of McLaren Macomb Hospital. MOC is a custom-built 40-foot motorhome that is a doctor’s office on wheels. It visits low-income suburbs of Detroit (MI), providing comprehensive and ongoing medical care for uninsured patients. Physician faculty, family medicine resident physicians, nurses, pharmacists, medical assistants, and medical and pharmacist students deliver wide-ranging care to those in need. The MOC staff offers a full spectrum of health care, including screenings, immunizations, acute care management, and treatment of chronic diseases, while also providing preliminary care for devastating health issues such as cancer. All medical treatments and procedures are at no cost to the patient. Medication, laboratory testing, and imaging are funded by the McLaren Macomb Foundation, the charitable arm of McLaren Macomb Hospital. If the MOC cannot meet a patient’s needs, they are referred to one of McLaren Macomb’s resident clinics or another McLaren Macomb physician.

## Case presentation

A 41-year-old G1P1 (C-section) Hispanic female immigrant from Central America presented to the MOC complaining of left breast pain gradually increasing in severity for approximately 3 months. This was accompanied by swelling, chills, and night sweats, along with skin changes and a self-detected mass.

She reported a previous episode 5 years ago, before her pregnancy, with pain and swelling in the left breast, resolving spontaneously. Three months before presenting to MOC, the patient had visited a physician (outside of MOC services) due to left breast pain and swelling. She was diagnosed with mastitis. She was prescribed meloxicam (7.5 mg/daily, orally) for inflammation and trimethoprim-sulfamethoxazole (TMP/SMX) 160 mg/800 mg (orally) twice a day for presumed bacterial origin. She was also on birth control pills (norgestimate 0.25 mg/ethinyl estradiol 35 μg).

The patient is a married stay-at-home mom who has no history of smoking or alcohol use. Her mother and father are both alive and healthy, and she has no family history of any cancers.

The following were noted on her admission: height 136 cm (4′5"); weight: 49.9 kg (110 lbs). Vital signs: heart rate 80 bpm; respiratory rate 14 bpm; temperature 36.8 °C (approx. 98.3°F); blood pressure 100/80 mmHg; pulse oximetry 100% on room air.

General presentation: alert and oriented; healthy appearing and in no acute distress. Eyes: pupils equally round and reactive to light, extraocular movement intact. ENT: no nasal erythema, rhinorrhea, or postnasal drainage. Neck: no thyromegaly, no palpable lymphadenopathy. Cardiology: regular rate and rhythm, no murmurs. Pulmonology: lungs clear to auscultation bilaterally. Abdomen: soft, non-tender, non-distended, regular bowel sounds. Vascular: good peripheral pulses, no edema. Neurology: cranial nerves 2–12 grossly intact, no gross neurologic deficits. Motor: Muscle strength 5/5 in all extremities.

**Fig. 1:**
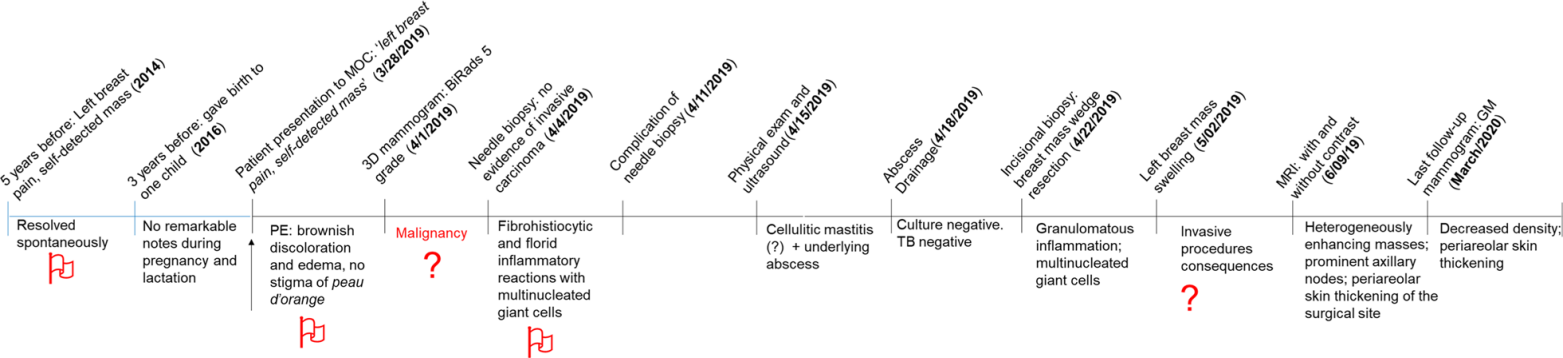
Key timeline information. The red flags point out the key facts to raise the question “what else it could be?” instead of malignancy. Dates of each event are enclosed in parentheses. Arrow (↑): the first time the patient presented to MOC. *MOC* Medical Outreach Clinic (McLaren Macomb Hospital), *PE* physical examination, *TB* tuberculosis

Breast: female chaperone present during the exam. Physical examination revealed a nodular mass in the outer quadrants of the left breast but currently without the presence of nipple discharge, fevers, or regional lymphadenopathy. Despite the presence of brownish discoloration and edema, the breast skin had no stigma of *peau d’orange* (timeline of the key information is summarized in Fig. 1).

Complete blood count, liver function tests, and renal function, urinalysis, serology, and lipid panel were within normal ranges.

A 3D mammogram and targeted left breast ultrasound were ordered and reported as a grade of BI-RADS [Breast Imaging-Reporting and Data System] 5, highly suspicious of malignancy. The patient was then referred to the McLaren Macomb General Surgery residency clinic. As the fastest and least invasive option, a percutaneous needle biopsy was agreed upon by the surgeon and the patient. The needle biopsy showed no evidence of invasive carcinoma, and instead showed fibrohistiocytic and florid inflammatory reactions with multinucleated giant cells.

The primary care team from MOC and the surgery clinic agreed that the needle biopsy result was inconsistent with the degree of abnormality seen on the patient’s left breast mammogram, ultrasound, and physical examination. Concerns for malignancy remained high. A collective decision was made; informed consent was obtained from the patient, and a full incisional biopsy was performed.

One week before the scheduled incisional biopsy, the patient's discomfort acutely worsened. She presented to McLaren Macomb’s emergency room (ER) complaining of discomfort, swelling, warmth, and redness at the site of the previous needle biopsy. Based on the physical exam and ultrasound, she was diagnosed with cellulitic mastitis with an underlying abscess. The surgical team was asked to see the patient in the ER, where incision and drainage of the abscess was performed, and a sample for culture was collected (anaerobes and Gram stain). Afterward, she was discharged from the ER with the same regimen of TMP/SMX (160 mg/800 mg, orally, twice a day) in addition to NSAID (meloxicam, orally, 7.5 mg/daily) as needed. Although the results of the culture were negative, she was kept on the antibiotic treatment regimen. In addition, diagnostic testing for tuberculosis (TB) was negative.

One week later, the patient presented to the MOC; the erythema and drainage from the site of the biopsy had improved. She was advised to continue with TMP/SMX. The patient was also seen by the General Surgery residency clinic team, who agreed that the cellulitis and its associated abscess had improved significantly.

An incisional biopsy of the original breast mass was performed at McLaren Macomb Hospital. A wedge resection of the left breast mass was performed along with multiple Tru-Cut biopsies. The remnant of the abscess initially treated during the patient’s ER visit was drained and thoroughly irrigated. A Penrose drain was installed. The patient tolerated the procedure well.

**Fig. 2:**
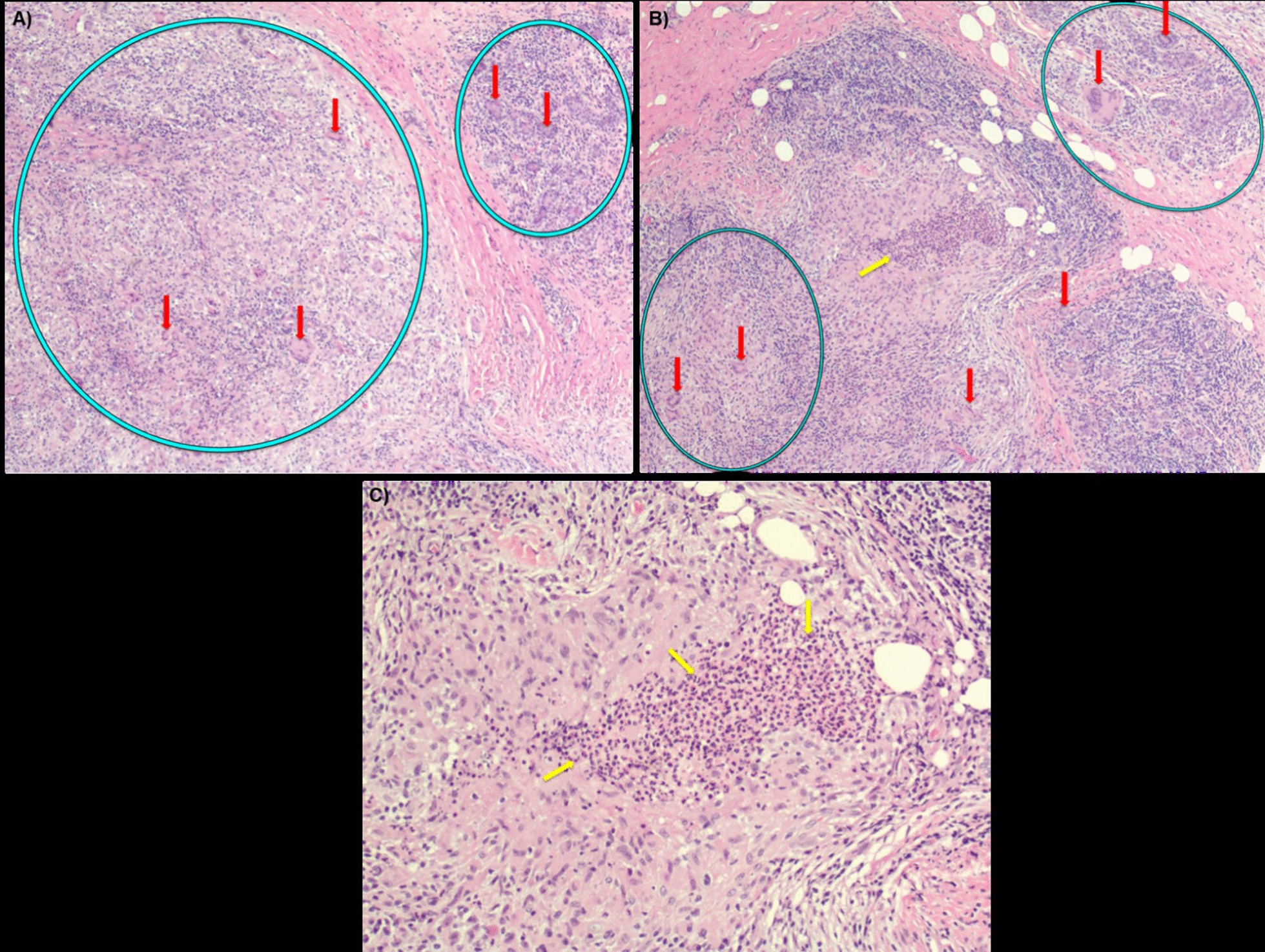
Breast biopsy. Photomicrograph of hematoxylin and eosin stain specimen. Green circles: granuloma (granulation tissue and granulomatous reaction: granulomatous inflammation). Red arrows indicate multinucleated giant cell. Yellow arrows show inflammatory infiltrate, mainly neutrophils. Panels **a** and **b**: magnification ×40; Panel **c**: magnification ×100

Evaluation of the patient’s multiple biopsies by a pathology team found granulomatous inflammation of the lobules with well-formed granulomas and multinucleated giant cells (Fig. 2). A diagnosis of cystic neutrophilic granulomatous mastitis was made. Periodic acid–Schiff (PAS) and Grocott's methenamine silver stain stains were negative for fungal elements. A special stain for Acid-Fast Bacilli revealed no organisms, with no bacterial organisms on Gram stain. These findings were discussed among the primary care physician, surgical, and pathology teams.

Three days postoperatively, the surgical team confirmed that the wound was healing well, and after 10 days the Penrose drain was removed. The patient continued on the same regimen of TMP/SMX. Three weeks post incisional biopsy, she was seen at the MOC experiencing a swollen tongue with a decreased taste sensation. A diagnosis of glossitis was made as a potential reaction to TMP/SMX; the medication was discontinued and changed to moxifloxacin, orally, 400 mg daily.

Due to the continued pain and swelling of her left breast mass, magnetic resonance imaging (MRI), with and without contrast, was ordered and revealed heterogeneously enhancing masses along with mildly prominent axillary nodes. The right breast and unaffected areas of the left breast showed a mild, nodular background. Periareolar skin in the area of the biopsy showed thickening, which corresponded to her surgical site, consistent with an inflammatory process. To decrease the patient’s pain and swelling from her breast mass, a 6-day taper of methylprednisolone (oral) was prescribed (Medrol dose pack). Three weeks after initiating the steroid regimen, she reported decreased pain and swelling, and on physical exam, the mass presented with a significant reduction.

The patient’s case was still under consideration and was presented to the tumor board of McLaren Macomb Hospital. Since the breast mass tenderness, swelling, and size had decreased, the consensus opinion was to hold on any further surgical intervention at that time.

At the next follow-up, the patient revealed that response to treatment had plateaued. The pain associated with the breast mass was still present and fluctuating. She reported she had been taking acetaminophen to manage the pain. Mild erythema and drainage were still present. Prednisone (40 mg/daily, 5 days) was prescribed along with moxifloxacin (400 mg/daily, 10 days).

One month later, the patient presented to the General Surgery clinic for follow-up, reporting a significant decrease in the breast mass size, and pain had nearly resolved. The following week, the patient presented to MOC experiencing nonpurulent yellow fluid discharge from the operative site. This was replicable during the physical exam, and the biological material was sent for culture. The patient was prescribed another short course of prednisone (20 mg/twice a day, 5 days). Cultures were negative, and the drainage quickly resolved. Ultimately the drainage was thought to be serous.

The patient’s follow-up mammogram 6 months later was consistent with the continued presence of cystic neutrophilic granulomatous mastitis (GM). This mammogram did show improvement over prior imaging, including a decreased density of the left breast mass along with the resolution of the surgical site of the periareolar skin thickening. Radiology graded the mammogram at BI-RADS 2 with the now known diagnosis of GM. The patient’s future care plan includes regular clinical follow-up at the MOC as well as mammography and breast MRI for monitoring of disease progression.

## Discussion/commentary

We present the complicated diagnosis and management of a patient with a rare breast disease presentation, granulomatous mastitis (GM). Although the history, physical examination, and needle biopsy flagged the patient’s findings as nonmalignant, the differential diagnosis became a critical element of care, and further workup was deemed necessary. This case report is unique in being conducted by a clinical team of health care workers offering services for underprivileged communities, as well as the difficulty in dealing with contradictory findings amid the lack of evidence-based medicine on GM. The conveyed message challenges the health care professionals to answer the decisive question of “What else it could be?” even when the initial presentation seems conclusive.

### GM and mastitis are commonly misdiagnosed

Since the clinical presentation of GM is consistent with infectious mastitis, most patients receive antibiotics at the beginning of their treatment. As the infection is presumed based on physical exam findings, antibiotic therapy is usually initiated without microbiological proof. GM is by definition a sterile inflammatory disease; therefore, antibiotic therapy usually fails [8, 9]. This would explain the lack of success with the initial pharmacotherapy.

Mastitis affects 3–20% of lactating women. Although mastitis can occur at any time during lactation, the majority of cases occur in the first 6 weeks [10]. The patient was not breastfeeding, so the inflammatory and infectious diseases resulting from lactation, usually observed in mastitis, were ruled out by our team at MOC. Also, it was remarkable that the patient reported a similar episode in her left breast roughly 5 years ago, prior to childbearing, which resolved spontaneously without medical attention. This fact lowered the suspicion of a tumor. However, the BI-RADS grade of 5 raised our concern for breast cancer. Because of the rarity of the disease and the lack of consensus guidelines for GM, the tumor board meeting was valuable for its discussion of further options for treatments and diagnostic modalities.

### GM considerations on mimicking breast cancer, cellulitic mastitis, and differential breast TB

In more than 50% of reported cases, the initial differential diagnosis of GM is malignancy or suspicion of breast carcinoma, and 15% of patients may present with regional lymphadenopathy [11], which was not a clinical manifestation in our current case.

The literature points out that the presence of *peau d’orange* may progress to ulceration; its presence is pathognomonic of cancer. [12] As it mimics breast cancer, GM is diagnosed by breast biopsy [7]. At the patient’s first visit to MOC, the absence of breast *peau d’orange* was documented, indicating that her condition might not be cancerous. In addition, the absence of carcinoma was confirmed by needle biopsy; however, because of the intrinsic limitations of this procedure, the severity of the clinical presentation, and the imaging reports, we concluded that a more definitive surgical procedure was necessary. The incisional biopsy confirmed the inflammatory process, compatible with cellulitic mastitis. GM is often initially misdiagnosed as cellulitis or furunculosis, and patients with GM may develop cellulitis, abscesses, and open draining sinuses [13, 14].

At this point, GM is our confirmed diagnosis. This reasoning is in accord with a multidisciplinary investigation conducted in Indiana reporting 12 times higher prevalence of idiopathic GM among Hispanic women immigrants from Central America than among non-Hispanic white women [15]. However, breast tuberculosis was also considered as a differential diagnosis in our patient, since she came from an endemic area [15] and presented with clinically suspicious breast lumps. Indeed, TB of the breast is an uncommon disease that is often difficult to differentiate from cancer of the breast when it presents as a lump [16]. The negative tests ruled out breast TB.

The optimal management of GM remains controversial [17, 18]. The main concern is the risk of recurrence observed for the different therapeutic approaches [19]. Antibiotics present the lowest efficacy in the treatment of mastitis in the absence of bacterial infection, with improvement rates ranging from 6 to 21% [20]. By comparison, corticosteroid therapy has a success rate of between 66 and 72%. In a metanalysis by Lei et al. [21], a pooled recurrence rate of 20% was reported for oral steroid therapy. Surgery alone or in combination with corticosteroids seems to have the lowest recurrence, with rates of 6.8 and 4%, respectively.

There are limited data in the literature and a lack of consensus on the use of antibiotic therapy for the treatment of GM [14]. Although the results were negative for a sample from abscess drainage, we decided to keep her on the empirical conservative therapeutic approach with TMP/SMX, following this urgent event. The antibiotic combination with an NSAID (meloxicam) had previously proven efficacious in controlling her pain. However, the patient developed glossitis, which was attributed to sulfonamide drug hypersensitivity reaction, and led us to change TMP/SMX to moxifloxacin. The drug reaction was confirmed by the absence of anemia or oral candidiasis, which are other common causes for glossitis [22], and its prompt regression once TMP/SMX was stopped.

The literature supports empirical therapy with antibiotics, anti-inflammatory agents, and surgery [14, 20, 21]. Although our patient’s responses were not optimal, the combination of surgical procedures, corticosteroids (methylprednisolone or prednisone), and antibiotics (the fluoroquinolone moxifloxacin was used) resulted in overall clinical improvement.

## Conclusion

The case is currently presented as idiopathic granulomatous mastitis (IGM) in a 41-year-old G1P1 patient. At the initial visit, she presented with multiple red flag warning signs (Fig. 1) that were concerning for advanced breast cancer. Despite presenting with a firm unilateral left breast mass associated with inflammation of the overlying skin, much of her history, physical, and clinical course were not consistent with breast cancer. There was no regional or generalized adenopathy or any *peau d’orange* despite her subsequent imaging studies continuing to confirm a tumor.

The history and physical exam are crucial to forming a strong and thoughtful differential diagnosis, which is a critical element of correctly identifying GM. The absence of both adenopathy and *peau d’orange*, along with the patient’s imaging results, led us to keep asking an important question, “What else could it be?” It is important to remain open-minded and flexible when considering a working differential diagnosis for a rare disease process.

Lessons learned:

- Clinical presentation and histologic data represent the cornerstone for differential diagnosis when advanced images are contradictory.
- Close follow-up with moderate-term therapy with TMP/SMX is essential due to the potential occurrence of adverse reactions, such as glossitis.
- The surgical procedure, antibiotic, and corticoid therapy is a successful combination to control the symptoms and progression of this rare benign breast disease.
- When multiple specialties manage a patient’s diagnosis, it is essential to have clear, concise, and frequent communication by all. This helps to ensure accuracy in the treatment plan and minimize unnecessary or duplication in testing.
- Patient compliance with follow-up and treatment regimens are equally important to a successful outcome as any other aspect of the treatment course.

In addition, the information from the present work goes beyond describing strategies for diagnosis, by depicting the interactions among a health care team working for underprivileged communities, which integrates both of the best multidisciplinary nonprofit services for the patient.

## Data Availability

Data sharing is not applicable to this article as no datasets were generated or analyzed during the current study.
